# The Infallible Microscope

**Published:** 1884-05

**Authors:** 


### THE INFALLIBLE MICROSCOPE!

At a recent meeting of the Philadelphia *Obstetrical Society*, Dr. William Goodell mentioned a number of cases in which experienced microscopists had given prognosis of early fatal termination, based on the cell formation of growths removed from the uterus; but the cases had *recovered*, and showed no evidence of any diseased condition!! The infallible microscope does occasionally err then!

We remember the case of a patient with a tumor on the back of thigh, which was pronounced a malignant growth by the microscopist at the University of Pennsylvania. The prognosis of this *learned* man, after quiet and careful microscopic examination, was that amputation at the hip joint, or death were the only alternatives. Amputation was performed and the patient died. Afterward it was discovered that the growth was not *malignant*, that amputation was not necessary, and *hence* that the patient's death was unnecessarily hastened. *Moral: do not trust too much to the microscope.*

